# Dr. James E. Culbert

**Published:** 1918-12

**Authors:** 


**Dr. James E. Culbert,** Niagara 1892, died Oct. 26. He was a member of the staff of Mercy Hospital.

